# Supplementary material for: Neutral competition boosts cycles and chaos in simulated food webs
Source: R Soc Open Sci. 2020 Jun 17;7(6):191532. doi: 10.1098/rsos.191532 (PMC7353966; doi:10.1098/rsos.191532)
Supplement: Online appendix [file rsos191532supp1.pdf]

A Online appendix

This is the Online Appendix for the paper:

Rodríguez-Sánchez P, van Nes EH, Scheffer M. *Neutral competition boosts chaos in food webs.*

A.1 Gottwald-Melbourne 0-1 test in a nutshell

The *0-1* test for chaos is designed for distinguishing between regular and chaotic dynamics in deterministic systems. It works directly with the observed time series, so a prior knowledge of the underlying dynamics is not required (as long as we know that they are deterministic). This short section is more a motivation than a rigorous proof. A minimal, geometrical approach to the method will be outlined. For a detailed, complete explanation please refer to (24).

The main input for the test is a one-dimensional time series of observations, ϕ_k, where the integer k represents the time index. This time series is used to build the functions of the parameter θ:

$$\begin{cases} p_n(\theta) = \sum_{k=1}^{n} \phi_k cos(k\theta) \\ q_n(\theta) = \sum_{k=1}^{n} \phi_k sin(k\theta) \end{cases} \tag{A.1}$$

The summands in equation A.1 are the horizontal and vertical components of a vector of length ϕ_k pointing in the direction $k\theta$. Consequently, each observation in our time series can be understood as the size of a step in the plane, being $k\theta$ its direction (see table 2).

k	0	1	2	3	...
$k\theta$	0	$\frac{\pi}{6}$	$\frac{2\pi}{6}$	$\frac{\pi}{2}$	...
$e^{ik\theta}$		$\frac{\pi}{6}$	$\frac{2\pi}{6}$	$\frac{\pi}{2}$	...
ϕ_k	2	1	0.5	0.25	...
$\phi_k e^{ik\theta}$					...

Table 2: Example showing a step by step geometrical construction of the elements inside the summation operator in equation (A.1). In this example we use a time series whose first elements are $\phi_j = \{2, 1, 0.5, 0.25, ...\}$. The parameter θ has been set to $\frac{\pi}{6}$.

Adding up the elements in table 2 as indicated by equation (A.1) can be interpreted geometrically as vector addition, i.e., performing one "step" after another (see figure A.1).

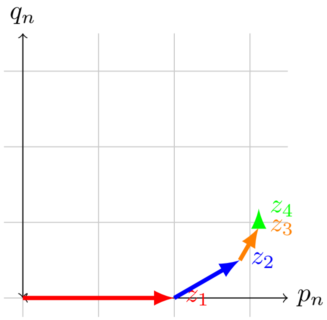

Figure A.1: Geometrical calculation of z_1, z_2, z_3 and z_4 for $\phi_j = \{2, 1, 0.5, 0.25, ...\}$ and $\theta = \frac{\pi}{6}$.

With this picture in mind, it is easy to understand the kind of paths that different types of time series will give rise to (see figure A.2). Constant time series generate cyclic paths (polygons) or pseudocyclic paths (polygons that do not close after a first round). Periodic or pseudoperiodic time series generate periodic or pseudoperiodic paths. Random time series generate brownian-motion-like paths. Provided that our system is deterministic, the apparent stochasticity of our path is a strong indicator of chaos.

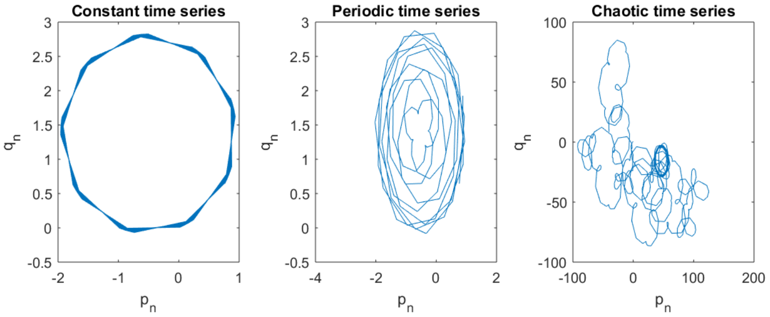

Figure A.2: First and second panels show the paths generated by the *0-1* test when applied to constant and periodic time series. The third panel shows the case with a chaotic time series (notice the different scale). While in the first two cases the paths remain inside a bounded domain, in the chaotic case the path drifts away from the starting point in a brownian-motion-like fashion.

The case of an underlying chaotic time series is the only one that generates a path that doesn't stay inside a bounded domain around the starting point (compare the third panel in figure A.2 with the other two). The *0-1* test uses the mean square displacement as a measure of this drift. The system is considered to be chaotic if the square displacement keeps growing for large times. If, on the contrary, it stays bounded, the test will consider the system not chaotic.

In the current manuscript, we used the time series corresponding to a non-extinct prey as input (ϕ_k) to the *0-1* test. Each time series was tested for 100 different values of θ, chosen from a uniform random distribution between $\frac{\pi}{3}$ and

$\frac{2\pi}{3}$. The median convergence was taken as statistic (this is done to exclude coincidental non-convergence if θ is close to the period of the system).

A.2 Results for species pools of different sizes

In the main body of the paper we focused our attention in families of food webs with species pools consisting of 12 prey and 8 predator species. In this section we show the results of the same analysis for food webs of different sizes.

A.2.1 Probability of chaos grouped by number of species

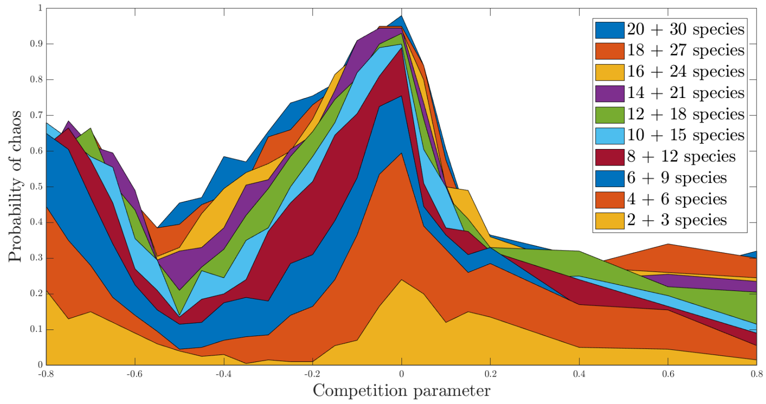

Figure A.3: Probabilities of chaos vs. competition parameter (ϵ) for the whole set of simulations. The estimated probability of chaos is represented on the vertical one. Each panel corresponds to an ecosystem with a different number of interacting species. The exact number is shown in each box, as number of predator + number of prey species.

A.2.2 Probability of each dynamical regime

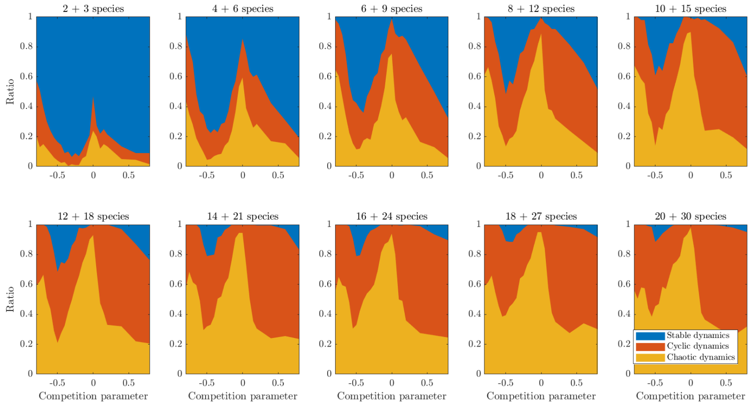

Figure A.4: Ratio of each dynamical regime vs. competition parameter (ϵ) for the whole set of simulations. The size of the species pool is shown in each box, as number of predator + number of prey species.

A.2.3 Biodiversity measurements

For each simulation, a biodiversity index was estimated as the number of prey species whose population was higher than a minimum threshold of 0.01 $mg\ l^{-1}$, averaged respective to time.

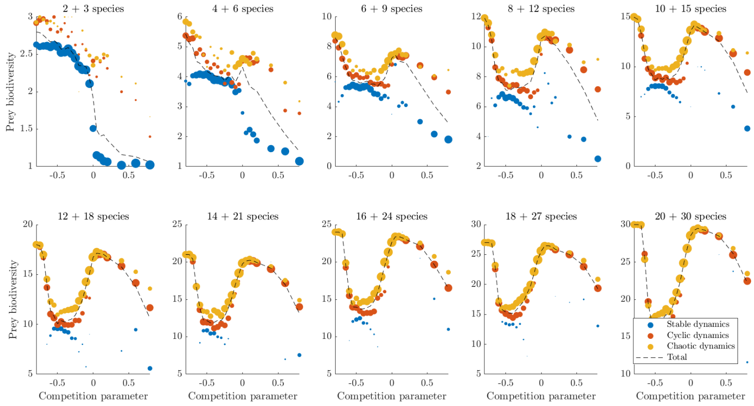

Figure A.5: Average prey biodiversity vs. competition parameter (ϵ). Each panel corresponds to a species pool of different size. For each value of the competition parameter (ϵ), 200 randomly drawn ecosystems were simulated. The dashed line shows the average number of prey species of these 200 simulations. The yellow circles represent the average prey biodiversity of those simulations who had chaotic dynamics. The red and blue circles represent the same for, respectively, cyclic and stable dynamics. The relative area of the circles represents the ratio of each kind of dynamics.

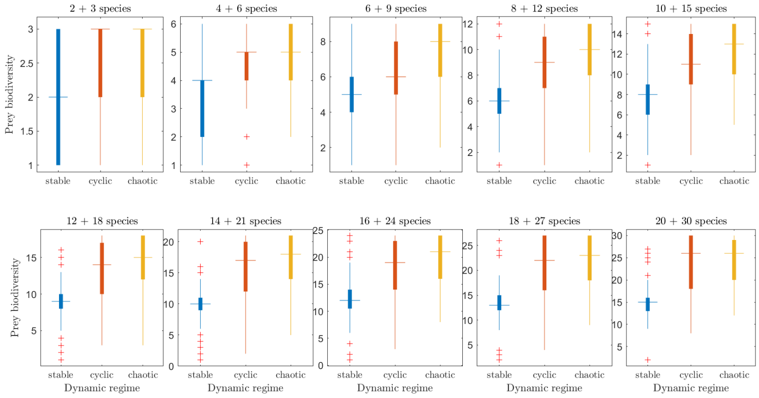

Figure A.6: Box and whisker plot of the prey biodiversity, after being classified as stable, cyclic or chaotic. The size of the species pool is shown in each box, as number of predator + number of prey species.

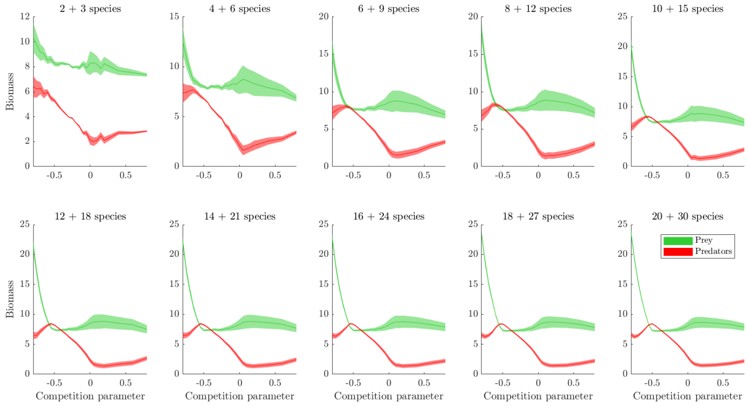

Figure A.7: Average biomasses grouped by trophic level vs. competition parameter (ϵ). The width represents standard deviation. The size of the species pool is shown in each box, as number of predator + number of prey species.

A.3 Effect of a neutral predation matrix

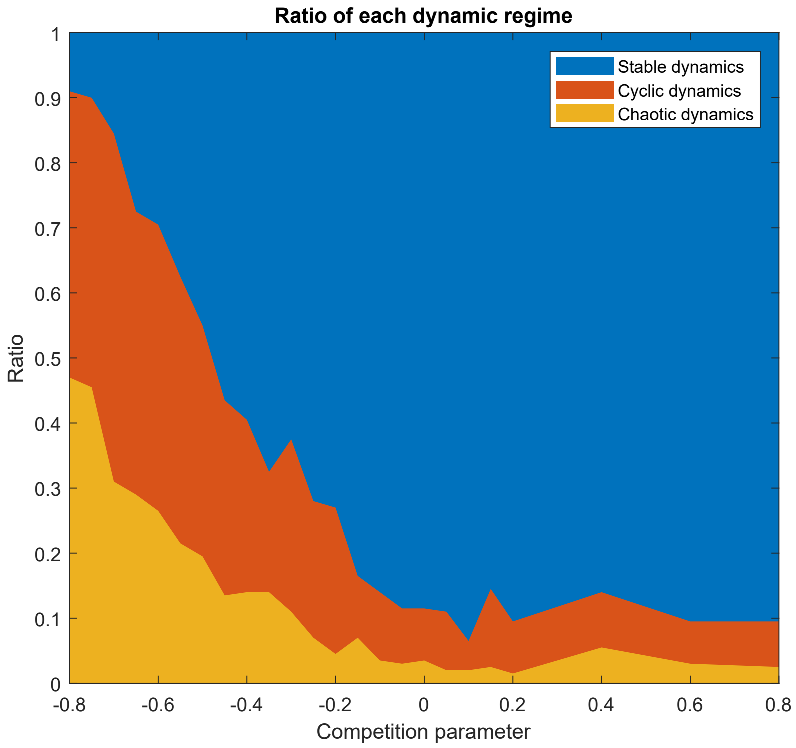

Figure A.8: Results for a system with 8 predators and 12 prey. The figure shows the ratio of each dynamical regime vs. competition parameter (ϵ). In this case all the elements in the predation matrix S have been set to 0.5. The predation parameters H and g have been drawn from uniform distributions bounded by $(1, 3)$ mg l^{-1} and $(0.3, 0.6)$ d^{-1} respectivelly.

A.4 Effect of the immigration term

In figure A.9 we show that our qualitative results remain true for different values of the immigration parameter f.

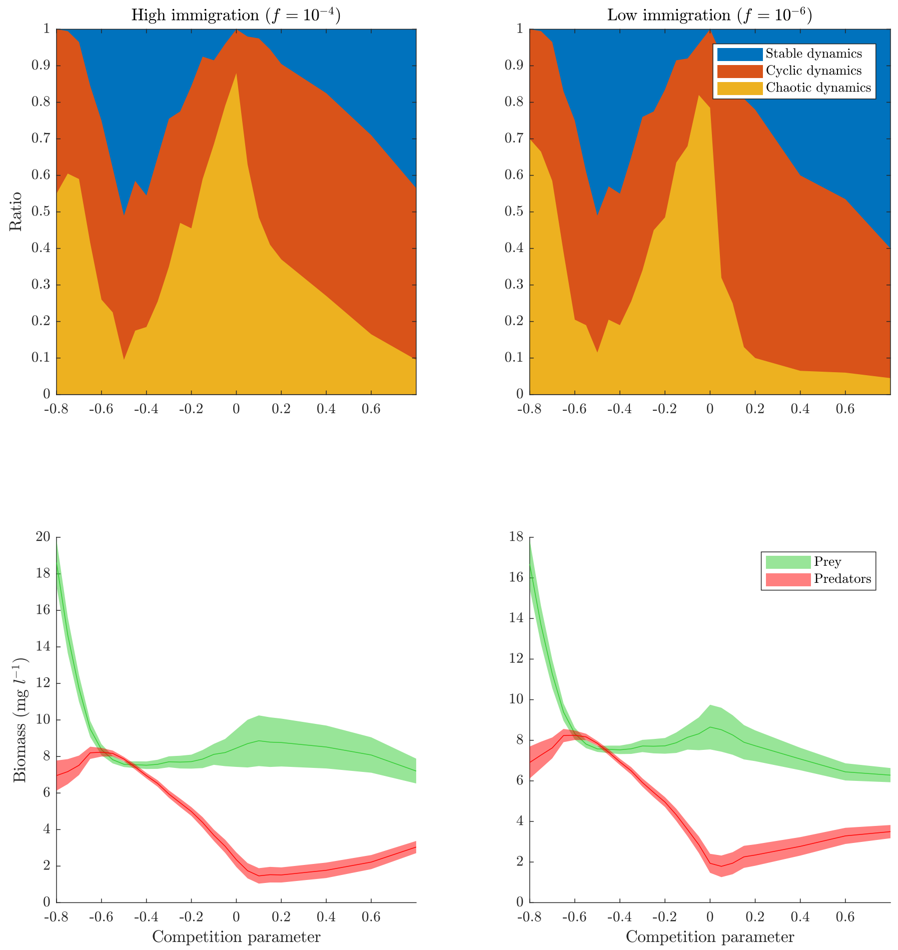

Figure A.9: Results for a system with 8 predators and 12 prey. The first and second columns have immigration rates f of 10^{-4} and 10^{-6} respectively. The upper row shows the ratio of each dynamical regime vs. competition parameter (ϵ) for the whole set of simulations. The lower row shows the average biomasses grouped by trophic level vs. competition parameter (ϵ). The width represents standard deviation.

The curious reader is invited to re-run our simulations using different sets of parameters. The code is available in the Zenodo repository (25).
